# Recent Pathophysiological Aspects of Peyronie's Disease: Role of Free Radicals, Rationale, and Therapeutic Implications for Antioxidant Treatment—Literature Review

**DOI:** 10.1155/2017/4653512

**Published:** 2017-07-04

**Authors:** Gianni Paulis, Gennaro Romano, Luca Paulis, Davide Barletta

**Affiliations:** ^1^Regina Apostolorum Hospital, Andrology Center, Albano Laziale, Rome, Italy; ^2^Castelfidardo Medical Team, Peyronie's Disease Care Center, Rome, Italy; ^3^Italian League against Cancer, Department of Urologic Oncology, Section of Avellino, Avellino, Italy; ^4^Faculty of Pharmacy, University of Rome “La Sapienza”, Rome, Italy; ^5^Department of Urology, Andrology Center, San Matteo Hospital, Pavia, Italy

## Abstract

Peyronie's disease (PD) is a chronic inflammation of tunica albuginea of the corpora cavernosa that causes an inelastic plaque resulting in penis deformation. Although its etiology is not completely known, there is general consensus that PD is genetically transmitted and secondary to penile trauma. In recent years, numerous studies demonstrated the role played by oxidative stress in PD pathogenesis, and other studies have described successful use of antioxidants in PD treatment. Oxidative stress is an integral part of this disease, influencing its progression. In the early stages of PD, the inflammatory infiltrate cells produce high quantities of free radicals and proinflammatory and profibrotic cytokines, with consequent activation of transcription factor NF-*κ*B. While conservative therapies commonly used in the early stages of PD include oral substances (Potaba, tamoxifen, colchicine, and vitamin E), intralesional treatment (verapamil, interferon, steroids, and more recently collagenase clostridium histolyticum-Xiaflex), and local physical treatment (iontophoresis, extracorporeal shock wave therapy, and penile extender), the significant results obtained by emerging treatments with the antioxidants cited in this article suggest these therapeutic agents interfere at several levels with the disease's pathogenetic mechanisms. Antioxidants therapy outcomes are interesting for good clinical practice and also confirm the fundamental role played by oxidative stress in PD.

## 1. Introduction 

Peyronie's disease (PD), a chronic inflammation of the tunica albuginea of the corpora cavernosa, causes formation of a hard, inelastic plaque, often resulting in penis deformation. Prevalence varies between 3.2% and 13%; it generally affects males around 50 years of age, but recently a rise in frequency has been recorded in younger patients [1, 2]. Like Dupuytren's contracture, PD more frequently affects white men, more rarely black, and Asian men [3]. Although its etiology is not completely known, there is general consensus that PD is genetically transmitted and secondary to penile trauma [4, 5]. Familiar aggregation and genetic transmission through HLA-B7 antigens had been amply described and understood [6–8]. Recently, a genetic locus that PD shares with Dupuytren's contracture was identified; WNT2 is the locus involved in genetic predisposition for both Dupuytren's disease and PD [9]. Previously, some studies had already observed the analogy and strong affinity between the two diseases: Dupuytren's contracture's typical cells with “cross-banded” nuclei had been observed in PD plaques in 1976; in 1989, Somers et al., after histological examination of PD plaques, proved an increase in type III collagen content, similarly to what occurs in Dupuytren's contracture [10, 11].

PD symptoms include penile pain (20%–70% of cases), penile curvature or deformation (94% of cases), erectile dysfunction (31.5% of cases), depressive state (48% of cases), and presence of a more or less palpable penile nodule [12–14]. Penile distortion occurs because the area of disease (plaque) has a tendency to gradually become less elastic; histological analysis of penile plaques unfailingly detects an alteration and disorganization of collagen associated with a reduction in elastin density [15].

The disease has three stages: acute posttraumatic period > inflammatory remodeling phase > stabilization phase.

The first stage of the disease is characterized by a brief acute posttraumatic period, lasting about 2 weeks, during which, after blood extravasation and fibrin and platelet accumulation, powerful recruitment of inflammatory cells, macrophages, and lymphocytes occurs [16]. During this brief period, no penile nodule is palpable and no plaque is visible on ultrasound. The next stage which lasts about 12–18 months is the inflammatory remodeling phase [16, 17]. In this stage various growth factors start to be produced, initially causing tissue degradation, production of a new extracellular matrix (ECM), and tissue remodeling; in this stage, rapid growth of plaque occurs, often associated with pain and penile curvature; the plaque then tends to gradually consolidate and become increasingly more fibrous, until one or more areas of calcification, which tend to grow larger, make their appearance. Pain (when present) then usually disappears, but an increase in curvature angle is almost always observed. At the end of this inflammatory phase, the disease stabilizes. In this third phase (stabilization), pain is typically absent, penis deformation is stable, and fibrocalcification of the plaque is practically complete; consequently, nodules are always clearly palpable in this phase.

### 1.1. Premises of Microscopic and Functional Anatomy

The tunica albuginea of the corpora cavernosa is made up of both collagen fibers (the majority) and elastic fibers. Whereas collagen fibers have greater resistance to traction and are inelastic, elastic fibers can be extended up to 150% of their normal length [18]. With no elastic fibers in the tunica albuginea, the penis would not be able to grow in length and size during erection. In normal conditions and in the absence of disease, the elastic fibers along the penile shaft, within the tunica albuginea, appear as a net interwoven with collagen fibers. However, in the penile crura and distal part of the corpora cavernosa (apex), the tunica albuginea is only or almost exclusively made up of collagen fibers [18, 19]. Thus, any local change in elastic fiber content can cause penile deformation.

The purpose of this article is to clarify and explain the significant role of the oxidative stress in PD pathogenesis; the other important objective of the article is to explain the resulting role of antioxidants in PD treatment.

## 2. Pathophysiology of Peyronie's' Disease (Figure 1)

### 2.1. Mechanism of Plaque Formation

Following trauma, delamination of the tunica albuginea occurs, with extravasation and accumulation of fibrin between the layers, due to insufficient activation of fibrinolysis [6]. Presence of the platelet thrombus and fibrin is responsible for the subsequent cascade of events. Fibrin acts as a strong chemotactic factor, recruiting inflammatory cells like neutrophil granulocytes, macrophages, mast cells, and so forth [20]. These cells immediately begin to produce proinflammatory cytokines, particularly transforming-growth-factor-beta-1 (TGF-ß1); platelets, too, along with the macrophages already present in the area after the posttraumatic extravasation, release TGF-ß1 cytokines and platelet-derived-growth-factor (PDGF) [17, 21]. Fibrin accumulation also promotes the influx of fibroblasts, already attracted by TGF-ß1 and PDGF [20, 21]. Macrophages and inflammatory cells are also responsible for the production of elastase, an enzyme that can cause degradation of the tunica albuginea's elastic fibers [20, 22]. Enhanced elastase activity and elastin-derived peptides act chemotactically, contributing to both the increase in the inflammatory cellular infiltrate and the influx of fibroblasts [20].

### 2.2. Role of the Disease's Most Important Biological Mediators 

#### 2.2.1. Transforming-Growth-Factor-Beta-1 (TGF-ß1)

TGF-ß1, produced by platelets, macrophages, neutrophils, and T-lymphocytes, is considered the most important profibrotic factor associated with PD.

TGF-ß1 possesses chemotactic activity on blood neutrophils, monocytes, lymphocytes, and fibroblasts [6, 17, 22–24]. TGF-ß1 activity includes the following: inducing fibroblasts to produce collagen; influencing ECM deposition, stimulating synthesis of individual components of the matrix, such as proteoglycans and fibronectin; promoting fibroblast proliferation and differentiation into myofibroblasts, that is, mesenchymal cells that share the same phenotype as smooth muscle cells and fibroblasts and therefore have the capacity both to contract and to synthesize collagen [6, 22, 23, 25]. In addition, TGF-ß1 has the following actions: induces* reactive oxygen species* (ROS) production; inhibits production of certain* matrix metalloproteinases* (MMPs), particularly MMP-1, MMP-8, and MMP-13, which have collagenolytic activity, increasing synthesis of* tissue inhibitors of matrix metalloproteinase* (TIMPs, specifically TIMP-1); increases synthesis of MMP-10, which can degrade elastin; activates the* nuclear factor kappa-light-chain-enhancer of activated B cells* (NF-*κ*B); inhibits fibrinolysis by inducing synthesis of* plasminogen activator inhibitor-1* (PAI-1); induces osteogenesis [17, 22, 26–32]. However, it has been shown that during the progression of disease the plaque undergoes calcification or ossification in 15%–25% of PD cases [32].

#### 2.2.2. Platelet-Derived Growth Factor (PDGF)

Produced mainly by platelets, but even by macrophages, PDGF, too, is profibrotic. Like TGF-ß1, PDGF has chemotactic activity on fibroblasts [6]. Furthermore, PDGF induces TIMP-1 synthesis, collagen biosynthesis, fibroblast proliferation, and differentiation into myofibroblasts, and it contributes to plaque calcification and ossification, also recruiting osteoblasts [6, 21, 22, 26, 33, 34].

#### 2.2.3. Interleukin (IL-1)

Proinflammatory cytokine IL-1 is produced by macrophages and fibroblasts [35, 36]. One of PD's numerous profibrotic factors is that it is strongly chemotactic for fibroblasts; it stimulates collagen synthesis; it increases fibroblast production of MMP-1, MMP-2, MMP-8, MMP-9, MMP-10, and MMP-13; it promotes synthesis of MMP-9 and MMP-3 and basic fibroblast growth factor; it stimulates production of* inducible nitric oxide synthase* (iNOS), an enzyme that plays a fundamental role in oxidative stress; it activates NF-*κ*B [6, 12, 17, 34, 35, 37, 38].

#### 2.2.4. Basic Fibroblast Growth Factor (bFGF)

This typically profibrotic growth factor is mainly produced by fibroblasts and myofibroblasts; it attracts fibroblasts in the area and induces their proliferation, stimulates collagen synthesis, increases synthesis of MMP-1 and MMP-9 (with elastic activity) and TIMP-1, and causes further local fibrin deposit [6, 33, 34, 37].

#### 2.2.5. Plasminogen Activator Inhibitor-1 (PAI-1)

Another important profibrotic factor produced mainly by platelets is that PAI-1 inhibits fibrinolysis, causing fibrin to remain at the site, triggering recruitment of inflammatory cells and subsequent release of profibrotic factors (cytokines, etc.); it also hinders collagenolysis and inhibits MMPs [39, 40].

#### 2.2.6. Tumor Necrosis Factor (TNF-*α*)

Produced by monocytes and macrophages, in the course of the disease its activity involves the following: recruiting fibroblasts and stimulating their proliferation; stimulating iNOS production; inducing cellular apoptosis; stimulating production of MMP-9 (elastase); activating nuclear factor NF-*κ*B; inducing PAI-1 synthesis [6, 34, 37, 41–43].

In PD, therefore, we have a preponderance of profibrotic factors causing excessive deposition of collagen and ECM; this leads to extreme disorganization of the collagen fibers, loss of elastic fibers, and consequent elasticity loss in the tissues involved. Although both type I collagen and type III collagen have been shown to be present in PD, the latter is much more plentiful in PD plaque [11, 30]. Microscopic studies have shown that besides an increased number of fibroblasts, a haphazard arrangement is present, with random orientation of collagen bands and a marked reduction in elastic fibers, which appear disrupted, with excessive accumulation of elastin [20]. Consequently, a thickening of the tissue (plaque) forms, resulting in elasticity loss and inevitable onset of penile deformation.

### 2.3. Free Radicals and Oxidative and Nitrosative Stress as Fundamental Component of Peyronie's Disease

In the last fifteen years, oxidative stress has been shown to play a decisive role in the physiopathological mechanisms of PD [6, 22, 26, 30, 32, 34, 35, 39].

At the disease site, ROS hyperproduction occurs in addition to cytokine production. The oxidative stress process begins very early, in the first 24–48 hours: through fibrin's chemotactic activity, an inflammatory cellular infiltrate is produced. Oxidation is truly triggered at this stage of the disease [30]. After leukocyte activation, neutrophil granulocytes and macrophages at the inflammatory site undergo degranulation and release lysosomal enzymes (collagenase, elastase, etc.); at the same time, a “respiratory burst” (or oxidative burst) occurs, which consists in rapid release of ROS (specifically, superoxide radicals and hydrogen peroxide) by neutrophils and macrophages [22]. The activation mechanism is supported by intracellular enzyme* nicotinamide adenine dinucleotide phosphate* (NADPH) oxidase, present in neutrophils, eosinophils, and macrophages. NADPH-oxidase catalyzes electron transfer from the cytoplasmic NADPH donor to molecular oxygen (acceptor), forming* superoxide anion* (O_2_^•−^) (Table 1). The inflammatory cells then produce high quantities of O_2_^•−^ that is dismutated to* hydrogen peroxide* (H_2_O_2_) thanks to the presence of the* superoxide dismutase* (SOD) enzyme (Table 1). SOD, a part of the endogenous antioxidant defense system, then intervenes as primary cellular defense against O_2_^•−^, causing catalytic removal of the superoxide radical. Extracellular SOD plays an essential role in maintaining the redox state, blocking negative local effects of O_2_^•−^. The superoxide anion also has a direct vasoconstrictive effect through mobilization of calcium ions and can therefore cause erectile dysfunction [44]. The existence of H_2_O_2_ produced via SOD, and simultaneous presence of metal cations such as iron (Fe^2+^), causes Fenton's reaction, which produces another highly toxic reactive species, the* hydroxyl radical* (HO^•^) (Table 1). However, H_2_O_2_ can also react with O_2_^•−^ through* the Haber-Weiss reaction* to produce HO^•^ (Table 1). H_2_O_2_ may also be transformed into* hypochlorous acid* (HOCl), given the presence of* myeloperoxidase* (MPO) in neutrophils and macrophages (Table 1). Subsequently, HOCl and H_2_O_2_ react to form* singlet oxygen* (^1^O_2_^•^), another highly toxic reactive species (Table 1). Production of large amounts of proinflammatory cytokines and ROS hyperproduction cause NF-*κ*B activation, a central event in the inflammatory response and further production of inflammatory cytokines [34, 35, 38]. NF-*κ*B, present in almost all cell types is a protein complex controlling DNA transcription; in PD, it regulates the expression of genes encoding for FGF, TGF-ß1, iNOS, fibrin, collagen, and so forth [30]. In the course of PD, the iNOS enzyme is mainly produced by macrophages, smooth muscle cells, and myofibroblasts [22]. Induction of an upregulation of iNOS therefore produces high local levels of* nitric oxide* and a series of its metabolites called* reactive nitrogen species* (RNS), which are free radicals like ROS but can cause greater cell and tissue damage [34]. In normal conditions,* nitric oxide* (NO) is the main mediator of penile erection and acts as a nonadrenergic and noncholinergic neurotransmitter, causing release of the smooth muscles of the corpora cavernosa. NO was also known as* endothelium-derived relaxing factor *(EDRF), and long-standing widespread prejudice held that EDRF could not be identified with unconventional gaseous molecule NO [45]. The chemical reaction producing NO occurs thanks to the presence of amino acid L-arginine via* nitric oxide synthase (NOS)* activity and leads to final synthesis of* L-citrulline* and NO (Table 1). The chemical process requires molecular oxygen and proceeds via synthesis of an intermediate, N*ω*-hydroxyarginine [46]. Furthermore, the presence of certain cofactors is necessary:* NADPH, flavin adenine dinucleotide* (FAD),* flavin mononucleotide* (FMN),* tetrahydrobiopterin (BH*_*4*_*),* and* calmodulin (CaM)* [34]. Whereas in normal conditions penile erection is mediated by the action of the two “constitutive” enzyme forms,* neuronal* (nNOS) and* endothelial NOS* (eNOS), which cause normal NO amounts to be produced, in pathological conditions and in PD high local* nitric oxide radical* (NO^•−^) concentrations occur, due to mediation of the inducible enzyme form (iNOS) [34]. While isoforms eNOS and nNOS are Ca^2+^- and calmodulin-dependent, iNOS is Ca^2+^- and calmodulin-independent and can be induced by inflammatory cytokines. NO^•−^ is synthesized by monocytes, macrophages, and fibroblasts. NO^•−^ production from iNOS activity is 100 to 1,000 times greater and has longer duration than NO^•−^ production from nNOS or eNOS [34]. It must be stressed that the effects of* nitric oxide radical* (NO^•−^) depend on its concentration, so when local NO^•−^ levels increase significantly (as in PD) a high oxidation state is produced. In these conditions, NO^•−^ starts to compete with SOD, removing* superoxide anion* (O_2_^•−^) and causing the formation of peroxynitrite (Table 1) [37]. Therefore, synthesis of peroxynitrite depends on the balance between O_2_^•−^ and SOD production and NO synthesis/consumption [34]. Peroxynitrite is a highly toxic, reactive molecule, which can cause cell damage (by lipid peroxidation and DNA fragmentation), tissue damage depletion of plasmatic antioxidants, endothelial smooth muscle relaxation impairment, vascular tone change, and organ dysfunction [34]. When peroxynitrite is protonated, peroxynitrous acid (HOONO) forms, another highly toxic reactive molecule which rapidly breaks down into* hydroxyl radical *(HO^•^) and* nitrogen dioxide radical* (NO_2_^•^), two other highly reactive cytotoxic molecules (Table 1). During the inflammatory process, some of these reactive species, such as HO^•^, can chemically react with lipids (lipid peroxidation) generating radicals, for example,* lipid alkyl radical* (L^•^),* lipid alkoxyl radical* (LO^•^), and* lipid peroxyl radical* (LOO^•^); these toxic substances can also react with NO^•−^ producing less reactive substances* (lipid nitrite/LONO*,* lipid peroxynitrite/LOONO)* which can play a role as diagnostic indicators of lipid peroxidation [30].

### 2.4. Mechanisms of Action of Antioxidants Used in the Treatment of Peyronie's Disease (Table 2)

#### 2.4.1. Vitamin E

This liposoluble substance was first suggested for PD treatment in 1948 by Scott and Scardino [47]. It has many properties. Antioxidant properties: vitamin E generally reacts with reactive species (R^•^), but its best-known reaction is with* hydroxyl radical *(HO^•^), with donation of a hydrogen ion (H^+^) and formation of a stable, no longer reactive molecule* (hydrogen peroxide) *[30]. Furthermore, when vitamin E reacts with* lipid peroxyl radical*, it leads to formation of a tocopheroxyl radical, a relatively stable, poorly reactive molecule that does not cause a new lipid peroxidation process (Table 1); the tocopheroxyl radical soon reacts with any available H^+^ molecule, going back to the stable form of vitamin E (*α*-tocopherol-OH). Vitamin E inhibits inflammatory cell ROS release (respiratory burst) [48].

*Antifibrotic Properties*. Vitamin E inhibits TGF-ß1 production; D-alpha-tocopherol and *α*-tocopherol succinate have been shown to inhibit cellular proliferation of fibroblasts in human pathological fibrosis [49, 50].

*Anti-Inflammatory Properties*. Vitamin E interferes with factor NF-*κ*B, also hindering proinflammatory cytokine transcription; vitamin E has an anti-COX-2 mechanism; it inhibits cell proliferation through a protein kinase C (PKC) inhibition mechanism [51–53].

Furthermore, vitamin E inhibits platelet adhesion and aggregation, improves endothelial function, and can repair DNA [54, 55].

*Literature Review*. Vitamin E is the oldest substance still currently in use in PD treatment. Scardino and Scott (1948-49) and Steinberg (1951) pioneered the use of vitamin E in medical treatment of PD [56, 57]. In their 1948 study, after treating 23 patients with tocopherol, Scardino and Scott obtained a plaque size reduction in 91% of cases, curvature improvement in 78% of cases, and resolution of pain in all cases. Pryor and Farell (1983) and Safarinejad et al. (2007) observed no significant improvement after treating PD patients with tocopherol compared to placebo treatment [58, 59]. However, several studies have shown that vitamin E is effective only when combined with other treatments (combination or multimodal therapy) [60–62]. In particular, our 2012 controlled study demonstrated the superiority of treatment results when adding vitamin E (600 mg/daily = group A) to a combination therapy (verapamil injection and iontophoresis + blueberries oral + propolis oral + topical Diclofenac/for 6 months = group B): (group A versus group B) success in reducing plaque size 97.14% versus 68.57% (*p* = 0.004); actual plaque size reduction 50.2% versus 35.8% (*p* = 0.027); penile curvature improvement 96.6% versus 48.4% (*p* = 0.0001); mean penile curvature decrease of 12.25° versus 6.73° (*p* = 0.01) [62].

#### 2.4.2. Carnitine

This molecule is very similar to an amino acid; in humans, it is synthesized in the liver, brain, and kidneys from essential amino acids lysine and methionine by ALC transferase. The carnitine system is comprised of L-carnitine, its esters (acetyl L-carnitine, propionyl-L-carnitine), and a complex enzymatic system located in the mitochondrial membrane. It has been estimated that carnitine produced endogenously is 1.2 *μ*mol per day per kg of body weight, but in omnivorous human beings about 75% of carnitine in the body comes from diet, while 25% comes from de novo biosynthesis [63].

Meat is the main external source of carnitine, but it is present in lower concentrations in cod and dairy and in even lower concentrations in plant-based products. Its main role is that of mitochondrial long-chain fatty acid transport; therefore it works to convert fat to energy.

*Antioxidant Properties*. It is scavenger and neutralizer of* superoxide anion*,* hydrogen peroxide*, and* peroxynitrite* [64].

L-Carnitine inhibits proliferation and osteoblastic differentiation of fibroblasts [65].

*Anti-Inflammatory Properties*. L-Carnitine is able to reduce blood levels of C-reactive protein (CRP), interleukin-6 (IL-6), and TNF-*α* [66]. It can inhibit factor NF-*κ*B, also suppressing* nitric oxide radical* production and iNOS protein expression [67]. The anti-inflammatory and antifibrotic properties of L-carnitine derive from its ability to cause upregulation of* peroxisome proliferator-activated receptor-γ* (PPAR*γ*) which can inhibit expression of proinflammatory cytokines (TGF-ß1, TNF-*α*, and IL-1) [68]. L-Carnitine also increases eNOS expression, contrasting the negative effects of* nitric oxide radical *and related* reactive nitrogen species* production (peroxynitrite, etc.) [69]. It is important to mention that L-carnitine is a peripheral antagonist of thyroid hormone action: L-carnitine inhibits triiodothyronine (T3) and thyroxine (T4) entry into the cell nuclei. A randomized study proved that 2 and 4 grams/oral/daily of L-carnitine are capable of reversing hyperthyroidism symptoms [70]. Therefore, if at therapeutic doses (<2 grams) there is no problem for euthyroid patients, hyperthyroid patients, or hypothyroid patients treated with thyroid hormones, caution should be exercised if patients to be treated with carnitine are hypothyroid. In case of long-term therapy with carnitine (at high doses) mild euphoria is possible as a side effect, as it negatively interferes with *γ*-aminobutyric acid (GABA), causing an antidepressant-excitatory effect.

*Literature Review*. Carnitine has been successfully used to treat PD. In their 2001 randomized controlled study, Biagiotti and Cavallini, after administering acetyl-L-carnitine (ALC) (1 g twice daily for 3 months) to patients with active-phase PD, compared results with a control group (tamoxifen 20 mg twice daily for 3 months): (ALC group versus tamoxifen controlled-group) erectile pain improvement 92% versus 50% of cases (*p* < 0.01); mean penile curvature decrease, 7.5° versus 0.5° (*p* < 0.01); plaque size reduction (mm2), 48.8 versus 26.9 mm^2^ (*p* < 0.01); inhibiting disease progression, 92% versus 46% [71].

In a subsequent controlled randomized study on patients with advanced resistant PD, Cavallini and Biagiotti (2002) administered propionyl-L-carnitine (PLC) (1 g twice daily) + 10 intraplaque infiltrations (one/week) with verapamil 10 mg for 3 months and compared results with those of the control group (tamoxifen 20 mg twice/day + 10 intraplaque infiltrations once weekly with verapamil 10 mg for 3 months); they demonstrated that the PLC + verapamil association (versus tamoxifen + verapamil control group) was able to significantly reduce both plaque volume (*p* < 0.01) and penile curvature (*p* < 0.01) [72].

#### 2.4.3. Pentoxifylline

PTX is a synthetic xanthine derivative structurally related to theophylline and caffeine. As hemorheologic agent, it was initially used to treat peripheral vascular diseases, cerebrovascular insufficiency, sickle cell disease, and diabetic neuropathy. PTX inhibits platelet aggregation and improves blood flow by increasing erythrocyte and leukocyte deformability [73].

*Antioxidant Properties*. They include inhibiting ROS release (respiratory burst) by neutrophils, inhibiting lipid oxidation, and interfering with oxygen-radical-mediated activation of proinflammatory transcription factor NF-*κ*B [74, 75]. Furthermore, PTX has been shown to have* hydroxyl radical* scavenging activity [76].

*Anti-Inflammatory Properties*. PTX reduces proinflammatory cytokines (TNF-*α*, TGF-ß1, and IL-1), NF-*κ*B, and COX-2 expression [77–79].

*Antifibrotic Properties*. Due mainly to its capacity to inhibit production of TGF-ß1, FGF, and PDGF [77–80], PTX can reduce collagen deposition, as well as having strong inhibitory effects on fibroblast proliferation, ECM synthesis, and myofibroblastic differentiation. Thanks to its capacity to inhibit TGF-ß1 and TNF-*α* production, PTX can also hinder production of PAI-1. PTX is also a nonspecific phosphodiesterase (PDE) inhibitor and therefore has further antifibrotic activity by reducing iNOS and collagen expression and stimulating fibroblast apoptosis within the tunica albuginea [81, 82].

*Literature Review*. Besides treatment of peripheral artery disease, PTX has been used multiple times in man to treat various inflammatory conditions associated with fibrosis (radiation-induced and pulmonary fibrosis, scleroderma, etc.). A 2003 article by Valente et al. described an experimental study which included PTX administration (10 mg/kg/day/oral in drinking water for 45 days) in rats with PD-like plaque elicited by injection of TGF-*β*1 into the penile tunica albuginea [82]. PTX monotherapy resulted in an 80%–95% reduction in both plaque size and collagen/fibroblast ratio. The authors concluded that PTX was effective in stimulating fibroblast apoptosis in the tunica albuginea and therefore suggested PTX may be effective in reversing PD fibrosis [82]. The first article in which PTX was proposed as treatment for PD was published in 2006 [83]. It was a report about a 51-year-old Caucasian patient suffering from PD, treated with PTX, who suffered from moderate erectile dysfunction associated with two calcifications of the corpora cavernosa and 30-degree penile curvature; after treatment with PTX 400 mg/three times daily for 6 months, curvature had decreased to about 10 degrees. The patient continued the same therapy for 2 more years; at follow-up the dorsal plaque had decreased in volume and consistency, erectile function had significantly improved, and ultrasound confirmed disappearance of dorsal calcification with persistence of ventral calcific plaque. In 2010, Lin et al. published in two articles appearing in the same journal, one shortly after the other, a study divided into two parts, concerning the effects of TGF-ß1 and PTX on cultures of samples of* tunica albuginea-derived fibroblasts* (TADFs) from men with and without PD; in particular, it focused on the effects on collagen metabolism, elastin expression, and elastogenesis [77, 78]. The specimens of plaque containing tunica albuginea were harvested from 12 patients with chronic PD, during surgery for curvature correction. Biopsy samples of healthy tunica albuginea were taken from 6 patients during surgery for penile prosthesis positioning. The study yielded the following results: TGF-*β*1 stimulates collagen and elastin production; treatment of TADF with PTX reduces the TGF-*β*1-mediated increase in elastogenesis and collagen deposition. In 2011, the first cohort study was published in which PTX was used to treat a group of 62 patients with PD and sonographic evidence of penile calcification [84]. The study's control group did not receive PTX. Treatment consisted in PTX 400 mg/three times daily (mean duration of treatment = 1.2 years). After treatment, patients were reassessed with a clinical exam and gray-scale or duplex ultrasound. Compared to the no-treatment group, patients who took PTX had a reduction in calcification volume (69.4% versus 33.3%, *p* = 0.03). Moreover, PTX-treated patients were much more likely to have stabilization (no change) or improvement in their calcium burden compared to patients of the control group (91.9% versus 44.4%, *p* < 0.001).

In 2012, a study assessed PTX efficacy in 74 PD patients using a combined therapy which included oral PTX (400 mg/three times daily) associated with oral L-arginine (1 g/twice daily) and verapamil injections (12 in total, every 2 weeks). Seventy-four PD patients were divided into two treatment groups: group I (39 patients), verapamil injections + oral therapy (L-arginine + PTX) + daily penile traction therapy (PTT) for 6 months; group II (35 patients), like group I but without PTT. Mean penile curvature improvement was 26.9° in group I versus 20.9° in group II. Although PTX was not used exclusively, the study demonstrated that combined treatment without PTT still led to significant penile curvature improvement [85]. In 2013, another report was published about a young PD patient who was treated successfully (significant plaque volume reduction) with a 2-year combined therapy (oral PTX/400 mg/3 times daily + oral tadalafil/5 mg/3 times a week + oral L-arginine/2500 mg/daily + oral PLC/250 mg/daily + oral vitamin B3/20 mg/daily + penile extender) [86]. Another report about exclusive use of PTX was published in 2014, describing PTX treatment in a 35-year-old PD patient with a large plaque in the distal third of the penis. Even though he had no bending, the patient complained of difficulty in coital penetration due to a lack of rigidity and tumescence in the terminal part of the penis. After oral PTX therapy (400 mg/oral/3 times daily) for 6 months, the patient had significant improvement of erectile function and glans tumescence, which made penetration possible [87]. In 2014, Alizadeh et al. published a study using PTX for treatment of PD. Ninety PD patients were randomized and divided into three treatment groups: group 1, oral PTX/1200 mg/day; group 2, verapamil injection/10 mg for up to 12 injections; group 3, oral PTX/1200 mg/day + intralesional verapamil injection. The outcomes were the following: (oral PTX versus intralesional verapamil versus oral PTX + intralesional verapamil) % of patients with plaque size reduction = 30 versus 33 versus 33; % of patients with improvement in penile curvature = 26.7 versus 36.7 versus 36.7; % of patients with improvement in erectile dysfunction = 46.7 versus 66.7 versus 86.7; % of patients with pain reduction = 73.3 versus 76.7 versus 80.0. Even in this study, PTX appeared effective in treating PD [88].

We published a controlled study last year in which PTX was used for 6 months in association with other antioxidant and anti-inflammatory substances for PD treatment [89]. All 307 patients of the study had PD in its evolutionary phase; the control group (group C, no treatment) included 101 patients.

The 206 treated patients were divided into two groups and differed only in whether they received penile PTX injections. Group A received PTX 100 mg 12 perilesional injections (twice a month for 6 months) + oral PTX 400 mg/twice a day + oral propolis 600 mg/daily + oral blueberry (*Vaccinium myrtillus* L., see below for a detailed description) 160 mg/daily + oral vitamin E 600 mg/daily + Diclofenac sodium 4% gel twice daily, as topical treatment for 6 months; group B received the same therapy as group A, but without PTX injections. The most significant results were as follows: (group A versus group B) % of patients with plaque size reduction = 100 versus 79.7 (*p* < 0.001); actual mean plaque size reduction = −46.9% versus −24.8% (*p* < 0.001); % of patients with improvement in penile curvature = 96.8 versus 56.4 (*p* < 0.001); actual mean penile curvature reduction = −10.1° versus −4.8° (*p* < 0.001); mean variation in % of penile curve angle = −40.7 versus −21.7 (*p* < 0.001); % of patients with complete disappearance of penile curvature = 6.3 versus 1.2 (*p* = 0.128); % of recovery of penile rigidity in patients with erectile dysfunction = 56.0 versus 23.5 (*p* = 0.005); % of patients with reduction in calcification size = 96.7 versus 70.8 (*p* = 0.01); actual mean reduction in calcification size = −54.0% versus −40.2% (*p* < 0.131); % of patients with complete disappearance of penile calcification = 9.6 versus 8.33 (*p* = 0.863). Incidence of side-effects after oral administration of PTX: blood pressure drop 0.46%, tachycardia 1.38%, dizziness 0.92%, skin rash 1.85%, headache 0.97%, hot flashes 0.97%, vomiting 0.48%, dyspepsia 2.42%, nausea 2.91%, meteorism 3.88%, and cumulative gastrointestinal adverse effects 9.69%. The only adverse effect of the penile PTX injection was local bruising at the infiltration site (3.57% incidence) [89]. The most important outcome was that PTX via perilesional penile injection was shown to be effective in treating PD and greatly statistically significantly improved the (already positive) results obtained with combined oral therapy alone. This study confirmed a peculiarity of PTX already mentioned in previous studies: a capacity to cause almost complete clearance of penile calcifications [83, 84].

#### 2.4.4. Coenzyme Q10 (CoQ10)

CoQ10, also known as ubiquinone, ubidecarenone, coenzyme Q, CoQ, or Q10, is a fat-soluble molecule involved in mitochondrial electron transport and oxidative phosphorylation and necessary for cellular respiration and* adenosine triphosphate *(ATP) production. The molecule exists in both reduced (ubiquinol, CoQ10H2) and oxidized (ubiquinone, CoQ10) form. CoQ10 inhibits increased ROS production, by blocking mitochondrial membrane depolarization. In its reduced form, CoQ10 acts as a potent endogenous antioxidant, interacting with oxygen-related radicals, hydrogen peroxide, and singlet oxygen and inhibiting lipid peroxidation (Table 1) [90]. CoQ10 prevents nitrosative stress by inhibition of excess nitric oxide radical production, and it participates in vitamin E and C regeneration [91, 92]. CoQ10 has a strong anti-inflammatory and antifibrotic activity, producing a decrease of proinflammatory cytokines TNF-*α*, IL-6, TGF-ß1, and* monocyte chemoattractant protein-1 *(MCP-1), one of the cytokines that regulate migration and infiltration of monocytes-macrophages; furthermore, higher MCP-1 expression was found in PD-fibroblasts [93]. In addition, CoQ10 decreases gene expression of IL-1*β*, NF-*κ*B, and iNOS [94].

*Literature Review*. A single study exists where CoQ10 was used to treat PD; this prospective, double-blind placebo-controlled randomized clinical trial demonstrated significant positive results: 186 patients affected by early chronic PD were divided into two groups of 93 patients each (treatment group and no-medication control group) [95]. Patients in the treatment group were treated with CoQ10, 300 mg/daily for 24 weeks. At the end of treatment, results were the following: (CoQ10 group versus placebo group) change in plaque size = −40.0% versus +35.7%; % of patients with objective curvature improvement = 54.3 versus 12.2; % of patients with increased curvature = 14.8 versus 69.5; % of patients with improvement of sexual function = 51.9 versus 8.6 [95].

#### 2.4.5. Propolis

Propolis has been used by mankind since ancient times for its curative properties. It is plant-based and consists in a mixture of compounds which bees* (Apis mellifera L*.) extract from tree bark and buds. The resinous substances extracted by the bees are then processed with the addition of wax, pollen, and saliva enzymes. The final product is a resinous mixture with a color varying in shade from yellow to black. Bees produce propolis to seal any small opening in their beehives, protecting themselves from the cold, rain, wind, and possible attacks by other insects. The most interesting property of propolis is that of preventing disease, protecting the bees in the beehive from parasites, bacteria, viruses, and microbes in general. Many polyphenolic compounds, flavonoids, phenolic acids (caffeic acid and cinnamic acid), and esters and fatty acids have been found in propolis. Flavonoids commonly contained in propolis are acacetin, apigenin, catechin, chrysin, galangin, kaempferol, luteolin, myricetin, naringenin, pinocembrin, quercetin, and rutin. Propolis also contains resveratrol (a natural stilbene derivative) and several minerals (Mg, Ca, I, K, Na, Cu, Zn, Mn, and F), as well as vitamins (B1, B2, B6, C, and E) [96]. Since propolis indirectly derives from plants, its composite content varies depending on the geographical position and climate of the area of production. For instance, the characteristic compounds of Chinese propolis are caffeic acid, caffeic acid phenethyl ester (CAPE), caffeic acid benzyl ester, 5-methoxy pinobanksin, pinobanksin, pinobanksin-3-O-acetate, pinocembrin, chrysin, and galangin [97]. Brazilian propolis contains the following: liquiritigenin, daidzein, dalbergin, isoliquiritigenin, formononetin, biochanin A, galangin, kaempferol, pinostrobin, and pinocembrin [98]. Geographical differences of propolis are significant. CAPE is present in amounts greater than 10 mg/g in propolis extracts from Australia, China (up to 29 mg/g), Hungary, New Zealand, Uruguay, and Uzbekistan. Less than 10 mg/g of CAPE is found instead in propolis extracts from Argentina, Bulgaria, Chile, Ukraine, and USA [99]. CAPE is scarcely present or even absent in propolis extracts from Brazil, South Africa, and Thailand. Poplars, and their buds in particular, are considered the main indirect source of propolis in Europe and North America, nontropical Asia, New Zealand, and North Africa (especially the Nile delta) [100]. Considering the heterogeneous nature of the composition of propolis, pharmaceutical industries use standard quantities of the individual compounds by chemical extraction, choosing the substances based on their particular properties.

*(i) Properties of the Most Common Substances Contained in Propolis*

*Galangin*

*Antioxidative Properties*. It affects lipid peroxidation and helps preserve other antioxidants such as vitamin E and vitamin C [101, 102].

*Antifibrotic Properties*. It diminishes gene expression of TGF-ß1, MMP-2, and MMP-9 [103].

*Anti-Inflammatory Properties*. It inhibits factor NF-*κ*B and reduces production of IL-1*β*, TNF-*α*, IL-6, and iNOS [104].

*Quercetin*

*Antioxidant Properties*. Quercetin decreases neutrophil activity (respiratory burst and degranulation) and inhibits release of NADPH-oxidase and MPO by decreasing generation of superoxide and derived ROS [105]. It is able to scavenge highly reactive species such as peroxynitrite, hydroxyl radical, superoxide anion, singlet oxygen, and lipid peroxyl radicals [106].

*Anti-Inflammatory Properties*. It inhibits production of TNF-*α*, IL-8, IL-1*α*, IL-1*β*, and IL-6 through inhibition of NF-*κ*B activation [107]. Moreover quercetin inhibits inflammatory enzymes cyclooxygenase (COX), lipoxygenase (LOX), and iNOS [108].

*Antifibrotic Properties*. It suppresses TGF-*β*-induced collagen production and inhibits matrix metalloproteinases (MMP-2 and MMP-9), which are normally inhibited by PAI-1 [109].

*Caffeic Acid Phenethyl Ester (CAPE)*. CAPE exhibits a stronger antioxidant activity than galangin [102]. It hinders oxidative burst and related polymorphonuclear neutrophil production of superoxide anion, hydrogen peroxide, and hypochlorous acid (HOCl) by activated leukocytes; moreover, it suppresses lipid peroxidation activity [110].

*Anti-Inflammatory Properties*. CAPE is a potent, specific inhibitor of NF-*κ*B-dependent transcriptional activity, as well as cyclooxygenase (COX-1 and COX-2) and lipoxygenase activity [110–112]. Moreover, CAPE inhibits iNOS, TNF-*α*, IL-1*β*, and IL-6 production [110, 111].

*Antifibrotic Properties*. CAPE attenuates TGF-*β*-mediated collagen synthesis, inhibits peroxynitrite-augmented TGF-*β*1 release, counteracts TGF-*β*-induced differentiation of fibroblasts into myofibroblasts, and the concurrent formation of collagen; it is capable of partially reversing myofibroblasts into fibroblasts and reversing collagen formation [112, 113]. 

*Chrysin*

*Antioxidant Properties*. It inhibits xanthine oxidase activity, which is responsible for superoxide anion production; it also inhibits the release of NADPH-oxidase and the oxidative burst of neutrophils [105].

*Anti-Inflammatory Properties*. It inhibits NF-*κ*B and iNOS enzyme activity and cyclooxygenase- (COX-) 2 and IL-1*β* gene expression, significantly inhibiting the release of NO, TNF-*α*, and related increase in PAI-1 [114, 115].

*Antifibrotic Properties*. It suppresses TGF-*β* and fibronectin expression; it also inhibits the profibrotic activity of PDGF [116, 117]. 

*Pinocembrin*. Pinocembrin possesses a high oxygen radical antioxidant capacity and nitrite scavenging capacity, inhibiting xanthine oxidase activity, which usually induces the generation of superoxide radicals; it also inhibits lipid peroxidation [118, 119].

*Anti-Inflammatory Properties*. It inhibits production of TNF-*α*, IL-1*β*, and IL-6, as well as iNOS and COX-2 expression [120].

*Antifibrotic Properties*. It inhibits TGF-*β* and MMP-2 and MMP-9 expression [121]. 

*Pinobanksin*

*Antioxidant Capacity*. It inhibits xanthine oxidase activity, which is responsible for superoxide radical production; it can also inhibit lipid peroxidation [118, 119]. Pinobanksin also has antiproliferative and proapoptotic activity [122].

*Apigenin*. Apigenin is an antioxidant; it inhibits xanthine oxidase activity responsible for ROS and hydrogen peroxide production.

*Anti-Inflammatory Activity*. It prevents migration of neutrophils toward the inflammatory site; it inhibits platelet aggregation, hindering the cyclooxygenase (COX) pathway; it also inhibits production of proinflammatory cytokines (IL-1*β*, TNF-*α*, IL-6, and IL-8) through inhibition of the activation of factor NF-*κ*B [123–125].

*Antifibrotic Properties*. It inhibits the expression of TGF-*β*1 and PDGF, therefore blocking collagen production; apigenin also inhibits differentiation of fibroblasts into myofibroblasts [125].

*Literature Review*. Cuban urologists (Lemourt et al., 1998) were the first authors to publish a study on the use of propolis in the treatment of PD, as they discovered by serendipity that propolis could cure the disease: they had noticed that a patient suffering from PD who had been treated with propoleum (oil of propolis) due to giardiasis had reported a “spontaneous” improvement in his penile curvature; they therefore began to administer propolis to several PD patients, subsequently publishing various studies reporting excellent clinical results [126–129]. In one of their studies, they analyzed the effects of propolis on 34 PD patients, assessing after 6 months two different daily doses of propolis (300 and 900 mg) in two respective groups (each made up of 17 patients) and concluded that patients treated with 900 mg/daily had superior clinical results; furthermore, clinical improvement and plaque volume reduction occurred earlier in this group [128]. In another study, the same authors studied the effects of propolis on PD in three treatment groups: (group 1) propoleum 900 mg/daily for over six months (10 patients); (group 2) 10 laser sessions every 2 months for over six months (8 patients); (group 3) propoleum 900 mg/daily + 10 laser sessions every 2 months (10 patients). The best outcomes in terms of curvature reduction were obtained in the group of patients treated only with propolis (mean penile curvature improvement: −10.8°); in the patients of the propolis + laser group, the curvature improvement was slightly inferior (−10.3°); only a few patients in the laser group referred to improvement (−8.0°) while a progression in curvature was observed in the remaining patients in this group, with a mean increase of 12.6°; as to plaque volume, mean US-measured reduction was 2.3 mm (only propolis group) and 12.16 mm (propolis + laser group); the laser group ultrasound results were not comparable due to the low number of patients compared to the other groups [129]. Another study where propolis was used to treat PD was published by Favilla et al. (2014); the authors used propolis in combination with other antioxidants and verapamil in patients with initial-phase PD, assessing in particular the efficacy of associating intralesional verapamil injection (10 mg weekly for 12 weeks) and oral antioxidants (group B) compared with verapamil injection monotherapy (group A). The oral antioxidant therapy consisted in a tablet/daily (for 3 months) with the following composition formula: propolis 100 mg, blueberry anthocyanins 80 mg, para-aminobenzoic acid 100 mg, Muira Puama 25 mg, soya isoflavones 50 mg,* Persea americana* 50 mg, vitamin E 36 mg, and Damiana 25 mg [61]. Although no significant differences were observed between the two groups in mean plaque size change (group A versus group B = −0.38 cm3 versus −0.29 cm3) and mean penile curvature improvement (−10.86° versus −11.97°), statistically significant results were obtained in group B in regard to orgasmic function (subdomain of 15-question International Index of Erectile Function, IIEF), intercourse satisfaction (subdomain of IIEF), overall satisfaction (subdomain of IIEF), and pain/visual analogue score (VAS) [61]. We ourselves have published five controlled studies in which we used propolis associated with other substances, always with excellent, statistically significant results; the most recent one of these studies is discussed in detail above in the section on pentoxifylline, and the others are in the following paragraph on bilberry [62, 89, 130–132].

#### 2.4.6. Bilberry (*Vaccinium myrtillus* L.)

Bilberry is also known as “European wild blueberry” and “Whortleberry.” Bilberry grows in forest undergrowth, generally in mountainous areas in North America and Northern and Central Europe. It contains numerous flavonoids, including anthocyanins, pigments that give fruit its color. Anthocyanins contained in bilberry are cyanidin, delphinidin, petunidin, malvidin, and peonidin; cyanidin is present in higher concentrations compared to the other anthocyanins (mean amount 0.053 *μ*g/mL); delphinidin and petunidin are present 2.5 times less, while malvidin and peonidin are present in very low concentrations (mean amount 0.011–0.012 *μ*g/mL) [133]. Although anthocyanins are the most preponderant substances in bilberry, it contains a variety of phenolic compounds, including quercetin, tannins, ellagitannins, and flavonols such as catechin, epicatechin, gallocatechin, and epigallocatechin (the latter two only in unripe fruit), and small quantities of vitamin C [134, 135]. Since it contains quercetin (already mentioned above when discussing propolis) bilberry has antioxidant, anti-inflammatory, and antifibrotic properties [105–109]. Due to its high anthocyanin content, bilberry has powerful antioxidant effects against superoxide anion, hydroxyl radical, and peroxynitrite [136]. Anthocyanins, especially delphinidin, quickly reacts with extended oxidation products of nitric oxide radical (RNS) [137]. Anthocyanins also reduce the levels of NF-*κ*B, TNF-*α*, and MCP-1, which regulates migration and infiltration of monocytes-macrophages [138]. Furthermore, delphinidin and cyanidin inhibit COX-2 and increase the protein level of eNOS; cyanidin is also able to cause downregulation of iNOS [139–141]. Anthocyanins can also inhibit phosphodiesterase (PDE) [142]. Bilberries (thanks to their high content of anthocyanins) also have antifibrotic action, suppressing TGF-*β*-induced collagen production [109]. Catechins contained in bilberries inhibit PDGF and FGF; moreover catechins show antiproliferative and proapoptotic activity [143].

*Literature Review*. In six controlled studies bilberry was used in combined PD therapy (see PTX and propolis sections above). Statistically significant results after treatment were found in all these studies [61, 62, 89, 130–132]. In particular, in our 2013 study, European blueberry was administered to 41 patients (group A) for 18 months, 160 mg/daily + verapamil 10 mg penile perilesional injections (twice monthly for 6 months and then once monthly for 12 months, for a total of 24 injections) + iontophoresis with verapamil 5 mg daily + propolis 600 mg/daily + vitamin E 600 mg/daily + topical Diclofenac 4%/daily. The control group (group B) was made up of 41 patients who received no treatment [132]. After 18 months of treatment, plaque size reduction occurred in 100.0% of patients in group A and none in group B (*p* < 0.0001); on the contrary, in group B plaque size always increased; in group A, mean plaque size reduction was −73.6%, while in group B, there was a mean increase in plaque size = +118.7% (*p* = 0.000); in group A, penile curvature improvement occurred in 81.5% of cases versus only 8.1% in group B; in group A, mean decrease of penile curvature angle was −16.74° while in group B it was −4.0° (*p* = 0.019) [132]. In the no-treatment group, penile curvature improved in only a few cases (8.1% = 3/41 cases); actually, in these cases the disease had progressed with an increase in plaque size which resulted in changes of the architectural structure of the penis that led to a paradoxical improvement in curvature.

## 3. Discussion and Conclusions 

As can be gathered from the number of references in our article, numerous studies have demonstrated the fundamental role played by oxidative stress in PD pathogenesis, and several studies have described successful use of various antioxidants in PD treatment. Oxidative stress is an integral part of the disease, influencing its progression; in the very early stages of PD, the inflammatory infiltrate cells begin to produce high quantities of ROS and proinflammatory and profibrotic cytokines, with consequent activation of transcription factor NF-*κ*B which induces iNOS production and subsequent release of high nitric oxide radical concentrations. The next chemical reaction cascade leads to production of high amounts of reactive nitrogen species, particularly peroxynitrite, which can damage cells and tissue by lipid peroxidation and DNA fragmentation. As described above, inflammatory cytokine- and NF-*κ*B-induced expression of iNOS leads to overproduction of nitric oxide radical, which reacting with superoxide anion causes peroxynitrite production [34]. Oxidative stress, furthermore, is an important factor in the possible onset of erectile dysfunction in PD, as hyperproduction of superoxide anion and peroxynitrite reduces nitric oxide concentration available for cavernosal muscle relaxation, resulting in long-term endothelial damage; superoxide anion is also reported to have a direct vasoconstriction effect [44, 144]. The presence of high iNOS concentrations has been proven in the cavernous tissue of men with PD [34]. The exact role of iNOS was long unclear, and a number of authors attributed a protective, antifibrotic role to it [39, 145]. Other authors, instead, proved that iNOS inhibition and peroxynitrite scavenging suppressed evolution of the inflammatory response and the normal course of collagen-induced diseases [146, 147]. Moreover, numerous studies listed in our literature review proved that iNOS inhibition after treatment with antioxidants (in PD patients) can have antifibrotic effects, resulting in significant clinical improvement [60–62, 71, 72, 82–89, 95, 128–132].

Since ROS damage occurs during the early stages of the disease, the treatment with antioxidants is only indicated in the initial stages of PD and not in the stabilization phase. For the same reason, the antioxidant therapy should be started immediately at the earliest diagnosis of the disease. Therefore, a “watchful waiting behavior” is not justified at the first time of PD diagnosis.

While conservative therapies commonly used in the early stages of PD include oral substances (Potaba, tamoxifen, colchicine, and vitamin E), intralesional treatment (verapamil, interferon, steroids, and more recently collagenase clostridium histolyticum-Xiaflex), and local physical treatment (iontophoresis, extracorporeal shock wave therapy-ESWT, and penile extender), the significant results obtained by emerging treatments with the antioxidants cited in this article suggest these therapeutic agents (which are also antifibrotic and anti-inflammatory) interfere at several levels with the disease's pathogenetic mechanisms (Table 2); combination therapy is therefore an effective treatment strategy today, capable of curing early-stage PD, yielding better results than those traditionally obtained with a single substance. Combination therapy outcomes are interesting for good clinical practice and also confirm the fundamental role played by oxidative stress in PD. However, further prospective randomized placebo-controlled studies are needed to evaluate the efficacy of emerging antioxidant therapies.

## Figures and Tables

**Figure 1:**
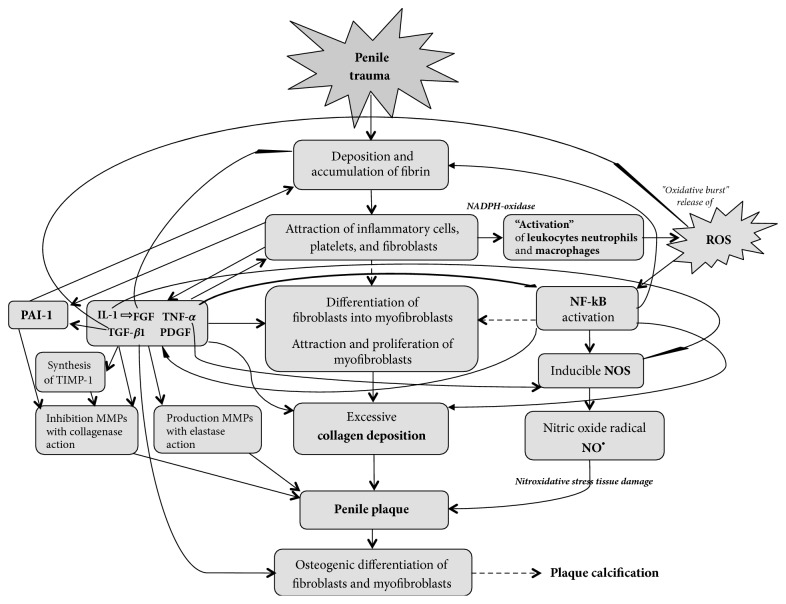
Pathogenetic mechanisms of Peyronie's disease.

**Table 1:** Chemical reactions during oxidative stress and direct reactions of vitamin E and coenzyme Q10 with reactive species.

Superoxide anion (O_2_^•−^)	NADPH + O_2_ → NADP^+^ + H^+^ + O_2_^•−^

Hydrogen peroxide (H_2_O_2_)	SOD
	2H^+^ + 2O_2_^•−^ → O_2_ + H_2_O_2_

Hydroxyl radical (HO^•^)	Fe^2+^ + H_2_O_2_ → Fe^3+^ + HO^−^ + HO^•^
	(Fenton's reaction)

Hydroxyl radical (HO^•^)	O_2_^•−^ + H_2_O_2_ → O_2_ + HO^−^ + HO^•^
	(Haber-Weiss reaction)

Hypochlorous acid (HOCl)	MPO
	Cl^−^ + H_2_O_2_ → HO^−^ + HOCl

Singlet oxygen (^1^O_2_^•^)	H_2_O_2_ + HOCl → H_2_O + Cl^−^ + ^1^O_2_^•^

Nitric oxide (NO^•^)	NOS
	L-Arginine + NADPH + O_2_ → L-Citrulline + NO^•^

Peroxynitrite (ONOO^−^)	O_2_^•−^ + NO^•^ → ONOO^−^

Peroxynitrous acid (HOONO)	ONOO^−^ + H^+^ → HOONO

Hydroxyl radical (HO^•^) and nitrogen dioxide radical (NO_2_^•^)	HOONO → HO^•^ + NO_2_^•^

Antioxidant activity of *vitamin E*(*α*-tocopherol-OH)	R^•^ (reactive species) + *α*-tocopherol-OH → RH + *α*-tocopherol-O^•^
	HO^•^ (hydroxyl radical) + *α*-tocopherol-OH → H_2_O + *α*-tocopherol-O^•^
	LOO^•^ + *α*-tocopherol-OH → LOOH + *α*-tocopherol-O^•^

Antioxidant activity of *coenzyme Q10*	Q10H2 + LOO^•^ → Q10^•−^ + H^+^ + LOOH

**Table 2:** Biological properties and mechanisms of action of antioxidants used in the treatment of Peyronie's disease.

Active substance	Biological properties	Mechanisms of action
Vitamin E	Antioxidant	(i) Scavenger activity against hydroxyl radical and lipid peroxyl radical
		(ii) Inhibition of respiratory/oxidative burst and relative release of ROS
	Anti-inflammatory	(i) Inhibition of NF-kappa-B activation and proinflammatory cytokine expression
		(ii) Inhibition of COX-2 activity
	Antifibrotic	(i) Inhibition of TGF-beta-1
		(ii) Inhibition of fibroblast proliferation
		(iii) Inhibition of PDGF activity
	Antiproliferative	(i) Inhibition of protein kinase C activity
		(ii) DNA repair activity
	Antiplatelet	(i) Antiadhesive and antiaggregating activity

Carnitine	Antioxidant	(i) Scavenger activity against super oxide, hydrogen peroxide, and peroxynitrite
		(ii) Suppresses iNOS protein expression and nitric oxide radical production
		(iii) Increase in eNOS expression (counteracting the negative effects of the nitric oxide radical)
	Anti-inflammatory	(i) Downregulation of C-reactive protein, IL-6, and TNF-*α* expression
		(ii) Inhibition of NF-kappa-B activation and proinflammatory cytokine expression
		(iii) Upregulation of PPAR*γ*
	Antifibrotic	(i) Inhibition of fibroblast proliferation
	Anticalcific	(i) Inhibition of osteogenic differentiation of fibroblasts

Pentoxifylline	Antioxidant	(i) Inhibition of respiratory/oxidative burst and relative release of ROS
		(ii) Hydroxyl radical scavenger
		(iii) Inhibition of lipid oxidation
		(iv) Downregulation of iNOS protein expression
	Anti-inflammatory	(i) Inhibition of NF-kappa-B activation and proinflammatory cytokine expression
		(ii) Inhibition of COX-2 expression
		(iii) Inhibition of TNF-*α* expression
	Antifibrotic	(i) Reduction of collagen deposition
		(ii) Inhibition of fibroblast proliferation
		(iii) Inhibition of myofibroblastic differentiation of fibroblasts
		(iv) Inhibition of TGF-beta-1 and PAI-1 expression
		(v) Stimulation of fibroblast apoptosis
		(vi) Nonspecific PDE inhibitory activity
	Antiplatelet	(i) Antiaggregating activity

Coenzyme Q10	Antioxidant	(i) Inhibition of increased production of ROS by blocking mitochondrial membrane depolarization
		(ii) Scavenger activity against hydrogen peroxide and singlet oxygen
		(iii) Downregulation of iNOS protein expression
		(iv) Inhibition of excess of nitric oxide production
		(v) Inhibition of lipid peroxidation
		(vi) Regeneration activity of antioxidants vitamin E and vitamin C
	Anti-inflammatory	(i) Inhibition of NF-kappa-B expression
		(ii) Downregulation of IL-6, TNF-*α*, MCP-1 protein, and IL-1-beta expression
	Antifibrotic	(i) Inhibition of TGF-beta-1

Propolis	Antioxidant	(i) Inhibition of release of NADPH-oxidase and myeloperoxidase (quercetin)
		(ii) Inhibition of xanthine oxidase activity responsible for superoxide anion production (chrysin, pinocembrin, pinobanksin, and apigenin)
		(iii) Inhibition of respiratory/oxidative burst and relative release of ROS (quercetin, CAPE, and chrysin)
		(iv) Scavenger activity against superoxide anion, hydroxyl radical, singlet oxygen, peroxynitrite, and lipid peroxyl radicals (quercetin)
		(v) Scavenger activity against superoxide anion, hydrogen peroxide, and hypochlorous acid (CAPE)
		(vi) Inhibition of lipid peroxidation (galangin, CAPE, pinocembrin, and pinobanksin)
		(vii) Regeneration activity of antioxidants vitamin E and vitamin C (galangin)
		(viii) Downregulation of iNOS protein expression (galangin, quercetin, CAPE, chrysin, and pinocembrin)
	Anti-inflammatory	(i) Inhibition of NF-kappa-B activation (galangin, quercetin, CAPE, chrysin, and apigenin)
		(ii) Inhibition of IL-1-beta and TNF-*α* expression (galangin, quercetin, CAPE, chrysin, pinocembrin, and apigenin)
		(iii) Inhibition of IL-6 expression (galangin, quercetin, CAPE, pinocembrin, and apigenin)
		(iv) Inhibition of IL-8 expression (quercetin, apigenin)
		(v) Inhibition of COX-2 (quercetin, CAPE, chrysin, pinocembrin, and apigenin)
		(vi) Inhibition of lipoxygenase (quercetin)
		(vii) Inhibition of neutrophil migration to the inflammatory site (apigenin)
	Antifibrotic	(i) Downregulation of TGF-beta-1 expression (galangin, quercetin, CAPE, chrysin, pinocembrin, and apigenin)
		(ii) Inhibition of MMP-2 and MMP-9 (both with elastase activity) expression (quercetin, galangin, and pinocembrin)
		(iii) Inhibition of myofibroblastic differentiation of fibroblasts (CAPE, apigenin)
		(iv) Capacity to reverse myofibroblasts into fibroblasts and reverse collagen formation (CAPE)
		(v) Inhibition of PDGF activity (chrysin, apigenin)
		(vi) Inhibition of PAI-1 and fibronectin expression (chrysin)
	Antiplatelet	(i) Antiaggregating activity (apigenin)

Bilberry (*Vaccinium myrtillus* L.)	Antioxidant	(i) Inhibition of release of NADPH-oxidase and myeloperoxidase (quercetin)
		(ii) Inhibition of respiratory/oxidative burst and relative release of ROS (quercetin)
		(iii) Scavenger activity against superoxide anion, hydroxyl radical, singlet oxygen, peroxynitrite, and lipid peroxyl radicals (quercetin)
		(iv) Scavenger activity against superoxide anion, hydroxyl radical, and peroxynitrite (anthocyanins)
		(v) Scavenger activity against nitrogen species/RNS (delphinidin)
		(vi) Downregulation of iNOS protein expression (quercetin, cyanidin)
		(vii) Increase in eNOS expression (delphinidin, cyanidin)
	Anti-inflammatory	(i) Inhibition of NF-kappa-B activation (quercetin, anthocyanins)
		(ii) Inhibition of IL-1-beta and TNF-*α* expression (quercetin, anthocyanins)
		(iii) Inhibition of IL-6 expression (quercetin)
		(iv) Inhibition of IL-8 expression (quercetin)
		(v) Inhibition of COX-2 (quercetin, delphinidin, and cyanidin)
		(vi) Inhibition of lipoxygenase (quercetin)
		(vii) Downregulation of MCP-1 expression (anthocyanins)
	Antifibrotic	(i) Downregulation of TGF-beta-1 expression (quercetin, anthocyanins)
		(ii) Inhibition of MMP-2 and MMP-9 (both with elastase activity) expression (quercetin)
		(iii) Inhibition of PDGF and FGB activity (catechins)
	Antiproliferative	(i) Antiproliferative and proapoptotic activity (catechins)
